# Milestones and Timescale of Poststroke Recovery: A Cohort Study

**DOI:** 10.1155/2020/8216758

**Published:** 2020-11-17

**Authors:** Marianna Loi, Alberto Zaliani, Marta Abbamonte, Elena P. Ferrari, Roberto Maestri, Luigi Trojano, Pietro Balbi

**Affiliations:** ^1^Department of Neurorehabilitation of Pavia via Boezio Institute, Istituti Clinici Scientifici Maugeri IRCCS, Pavia 27100, Italy; ^2^Department of Neurorehabilitation, Casa di Cura Figlie di San Camillo, Cremona 26100, Italy; ^3^Department of Neurorehabilitation, IRCCS Fondazione Don Carlo Gnocchi, Milano 20100, Italy; ^4^Biomedical Engineering Unit of Montescano Institute, Istituti Clinici Scientifici Maugeri IRCCS, Montescano 27040, Italy; ^5^Department of Psychology, University of Campania “Luigi Vanvitelli”, Caserta 81100, Italy; ^6^Department of Neurorehabilitation of Telese Terme Institute, Istituti Clinici Scientifici Maugeri IRCCS, Telese Terme 82037, Italy

## Abstract

**Background:**

Progressive increase of an aging population in Western countries will result in a growth of stroke prevalence. As many stroke survivors chronically show severe disability, increase in economic, social, and medical burden could be expected in the future. Objective and subjective measures of poststroke recovery are necessary to obtain predictive information, to improve the treatments, and to better allocate resources.

**Aim:**

To explore a measure of the temporal dimension of poststroke recovery, to search for predictive association with multiple clinical variables, and to improve tailoring of poststroke treatments.

**Method:**

In this observational monocentric cohort study, 176 poststroke inpatients at their first cerebrovascular event were consecutively enrolled. A novel measure based on the time needed to reach the main milestones of motor recovery was proposed. Moreover, two commonly used outcome measures, a measure of global functioning (Functional Independence Measure (FIM™)) and a measure of neurological poststroke deficit (Fugl-Meyer scale), were collected for the investigations of possible predictors.

**Results:**

The patients showed a substantial increase in Fugl-Meyer and FIM scores during the rehabilitative treatment. The acquisition of three milestones was significantly associated with female sex (autonomous standing), length of stay and Fugl-Meyer initial score (autonomous walking), and Fugl-Meyer initial score (functional arm). These findings provided quantitative data on motor milestone reacquisition in a sample of poststroke patients. It also demonstrated the value of the Fugl-Meyer score in predicting the acquisition of two motor milestones, relevant for daily life activities.

**Conclusion:**

Systematic recording of the timescale of poststroke recovery showed that motor milestone reacquisition happens, on average and when attainable, in less than 30 days in our sample of patients. The present study underscores the importance of the Fugl-Meyer score as a possible predictor for better improvement in reacquisition times of milestone functional recovery.

## 1. Introduction

According to a recent systematic review on global burden of diseases [1], in 2015, neurological disorders globally ranked as the leading cause group of disability-adjusted life years (DALYs) (250.7 per million, comprising 10.2% of global DALYs) and the second leading cause group of deaths (9.4 per million, comprising 16.8% of global deaths). Globally, stroke remained the main contributor to this burden (47.3% of DALYs and 67.3% of deaths).

Worldwide, from 1990 to 2015, the DALY (a measure of overall disease burden, expressed as the number of years lost due to illness, disability, or early death) for all neurological disorders increased by 7.4%. Increasing life expectancy in recent years and improved treatments for the acute phase of neurological illnesses have been usually considered responsible for such a trend.

In industrialized countries, a progressive shift in health care systems is being developed from acute care settings towards prevention and treatment plans mainly addressing chronic illnesses [2].

Rehabilitation treatments have the potential to reduce the disability in patients experiencing poststroke sequelae [3–5]. Yet, the field of rehabilitation still lags well behind the recent progresses of the acute treatment of stroke, and suggestions have been recently provided to reduce the gap [6, 7]. Among those, it is important to develop novel timeframes and measures to examine outcomes, able to overcome the lack of knowledge about biology of recovery and its time course.

Here, we reappraise and propose functional measures of the poststroke outcome, which rely on reaching the main motor milestones of the poststroke recovery. The main aim of the study is to exploit these measures to find out predictive association with clinical variables.

In addition, we aimed to report on the timescale and pattern of poststroke recovery, as they can be a useful tool to describe and compare the results of the rehabilitation treatments, to provide an evidence-based empirical outcome metrics, and to aid in shaping and settling the most favourable and rational resource assessment in poststroke care.

Data on the timescale of motor milestone recovery have been acquired in few previous studies [8–12]. In general, past studies tried to associate the recovery of selected milestones (which varied among the studies and often were broadly defined) with anatomical location and size of the cerebral lesions, or with different types of clinical or neuromotor assessment.

No agreement exists in the literature on the optimal set of motor milestones to be recorded during poststroke recovery. Some authors selected up to sixteen milestones [8], but most studies recorded three to four milestones with major functional relevance. We chose eight motor milestones easily detectable by physiotherapists in the setting of the rehabilitative sessions. The items were selected among gross body movements also evaluated in previous studies, which are central to the disability related to the stroke and represent milestones of recovery when regained [8–12]. Among those, we performed more detailed analyses on those three with major functional relevance in terms of disability during daily activities and aid workload for care givers: autonomous standing, autonomous walking and functional arm. The three motor milestones were selected also due to their importance in characterizing motor function recovery after stroke [13, 14]. Autonomous standing, indeed, is mainly involved in the ability to perform bed to chair transfers with minimal or no assistance, autonomous walking, even along short distance and with orthoses, usually makes the patient able to get about independently at home, and functional arm can provide a valuable degree of independence in eating and personal care.

We apply the proposed measures in a monocentric cohort study on a group of *de novo* stroke patients who went through an inpatient rehabilitation treatment. Common poststroke outcome measures are also reported, alongside with those newly proposed, and association statistics with demographic and clinical variables are searched for.

The results of the present study reappraise the value of the Fugl-Meyer score [15] in predicting the acquisition of two motor milestones with major functional relevance after a rehabilitation treatment and provide quantitative data on the timescales and percentage of the poststroke milestone acquisition.

## 2. Materials and Methods

The study design and protocol were approved by the institutional review board and by the Ethics Committee of ICS Maugeri IRCCS Pavia (PV) and were in accordance with the World Medical Association's Code of Ethics (as per the 1967 Declaration of Helsinki). On admission, all patients signed a consent form for the authorization of access to their medical records; all patient data were managed anonymously.

This observational study was performed according to the guidelines of the STROBE (STrengthening the Reporting of OBservational studies in Epidemiology) Initiative.

**Figure 1:**
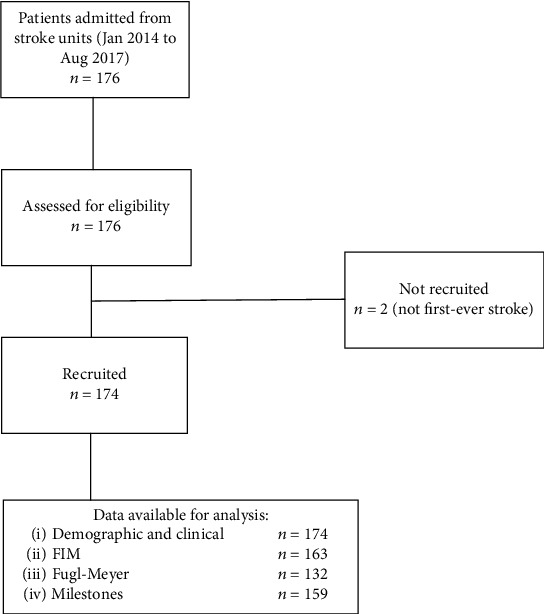
Flow diagram.

The study population consisted of 176 patients consecutively admitted for stroke rehabilitation from January 2014 to August 2017 to the inpatient neurological rehabilitation unit of the Scientific Institute of Pavia via Boezio (Istituti Clinici Scientifici Maugeri IRCCS), which has a regional user base and is certified (ISO9001, quality management systems) for activities of rehabilitation (Figure 1). Patients were referred to the rehabilitation unit from the local stroke unit in the city of Pavia and were included in the study if (i) they were at their first ischaemic or hemorrhagic stroke and (ii) the stroke occurred less than 30 days before admission. The exclusion criteria were as follows: (i) time of admission longer than 30 days from the stroke, (ii) patients with previous cerebrovascular events, and (iii) patients with previous neurological diseases other than stroke. Two of the 176 enrolled patients were excluded from the study, due to previous cerebrovascular events detected on close scrutiny after enrolment.

The number of the referred patients during the study period determined the sample size.

The multidisciplinary stroke rehabilitation team comprises the following professionals with expertise in poststroke rehabilitation: neurologist, physiatrist, physiotherapist, occupational therapist, speech and language therapist, neuropsychologist, and nurse. The patients received physical and occupational therapy (including passive and active joint mobilization, stretching, and coordination exercises) for 3 hours per day for 5 days and for 1 hour for 2 days (weekends) of each week. Patients with more severe initial disability were managed with lower intensity programs. Admission and discharge Functional Independence Measure [16] (FIM™) and Fugl-Meyer [15] scores were recorded by physicians or trained therapists, as a part of the formal rehabilitation program. Each patient was rated by the same examiner (M.L., M.A., or E.P.F.) along the study.

In addition, we designed a further outcome measure based on the percentages and times of acquisition of the main milestones of the poststroke motor recovery, derived from previous studies on motor milestone acquisition [8–12], and on their relevance in characterizing motor function recovery after stroke [13, 14]. In particular, we recorded the time of recovery of the following eight functional and motor milestones:

- Muscle tone recovery, which is defined as the increase in muscle tone during passive mobilization on one of the affected limbs after the initial (if occurred) flaccidity and tested in comparison to the not affected limb; if no flaccidity develops after stroke, a time recovery score of 0 is assigned
- Trunk control, defined as the ability of the patient to maintain an unsupported sitting posture for longer than 1 minute at bed or plinth height to allow hips, knees, and ankles positioned at 90°, with both feet flat on the floor and weight distributed evenly between the ischiatic tuberosities
- Assisted standing, defined as the ability of the patient to maintain a standing posture for longer than 1 minute, with the therapist assisting in avoiding occasional failures of the affected leg to sustain the body weight
- Autonomous standing, defined as the ability to maintain an unsupported standing posture for longer than 1 minute without assistance, even with orthoses (canes, walkers, etc.), and physical help is permissible in making the transition from sitting to standing, but there was no help during the standing period
- Assisted walking, defined as the ability to walk for ten meters or more, with or without orthoses, with the help by the active assistance of the therapist (correcting for balance, helping in moving forward the affected leg, etc.)
- Autonomous walking, defined as the ability to walk without assistance, with or without orthoses, for ten meters or more in a straight line
- Active motility of the affected arm, defined as the recovery of spontaneous motility of the affected arm, even with synkinesis
- Functional recovery of the affected arm, defined as the ability to perform simple functional movements with the affected arm (for example, to bring something to the mouth)

The number and percentage of patients who actually achieved each milestone were recorded, and the time in days to achieve each milestone was recorded for those patients who actually reached those milestones.

Data were collected along the stay of patients in the unit. FIM and Fugl-Meyer scales were administered at both admission and discharge; data on milestone recovery were collected on a daily basis by the physiotherapist as part of the rehabilitation treatment.

The concurrent diseases of the patients were also collected at admission, as well as the Cumulative Illness Rating Scale for Geriatrics [17, 18] (CIRS-G) and the modified Barthel Index [19, 20] (Mahoney and Barthel, 1965; Shah et al., 1989) scores.

### 2.1. Statistical Analysis

We computed descriptive statistics for all variables collected at the study entry and for changes in the Fugl-Meyer score and FIM score between the study entry and discharge.

Two separate multiple linear regression models were computed, which simultaneously included all demographic and clinical variables collected at the study entry (age, sex, length of stay in the unit, Fugl-Meyer score, FIM score, CIRSGpt, CIRSGig, and modified Barthel score) as independent variables and adopted FM and FIM as dependent variables, respectively.

Moreover, we analysed the achievement of the milestones and the time needed for achieving them. Last, we ran separate multivariable binary logistic regression models (one for each of the outcome measure) to comprehend the independent relationships between the variables collected at the study entry (age, sex, length of stay in the unit, Fugl-Meyer score, FIM score, CIRSGpt, CIRSGig, and modified Barthel score) and three main outcome measures at discharge (autonomous standing position, autonomous walking, and functional recovery of the paretic arm), codified dichotomously with the same criteria (0 if absent and 1 if present). The three measures were chosen among the others due to their relevance in achieving a fairly good independence in daily activities and because they could provide an indirect comparison with previous works [8–12].

## 3. Results

Baseline features of the patients and changes in FIM and Fugl-Meyer scores from admission to discharge are reported in Table 1. The mean FIM and Fugl-Meyer gains were 25.8 ± 17.1 and 22.0 ± 33.8 points, respectively.

The multiple linear regression ran for understanding predictors of changes on scales of the functional outcome did not provide a significant model either for changes on the Fugl-Meyer scores (*R* = 0.229, squared *R* = 0.053, *F* = 0.959, *p* = 0.464) or for changes on the FIM score (*R* = 0.281, squared *R* = 0.079, *F* = 1.782, *p* = 0.095), but we observed a significant univariate correlation between duration of rehabilitation stay and changes on FIM score, albeit with a small amount of explained variance (*R* = 0.246, squared *R* = 0.060, *F* = 10.458, *p* = 0.001).

The features of the observed recovery milestones are globally reported in Table 2. In our sample, muscle tone recovery, trunk control, assisted standing, and active motility of the affected arm were already present in a majority of patients on admission (12 days after stroke onset, on average). Autonomous standing, autonomous walking, and functional arm recovery milestones were acquired during the rehabilitation treatment in 57.9%, 69.2%, and 40.9% of patients, respectively. Autonomous walking, autonomous standing, and functional arm were not reached in 23.9%, 14.5%, and 12.6% of patients, respectively. Less than 10% of our sample did not reach the remaining milestones.

**Figure 2:**
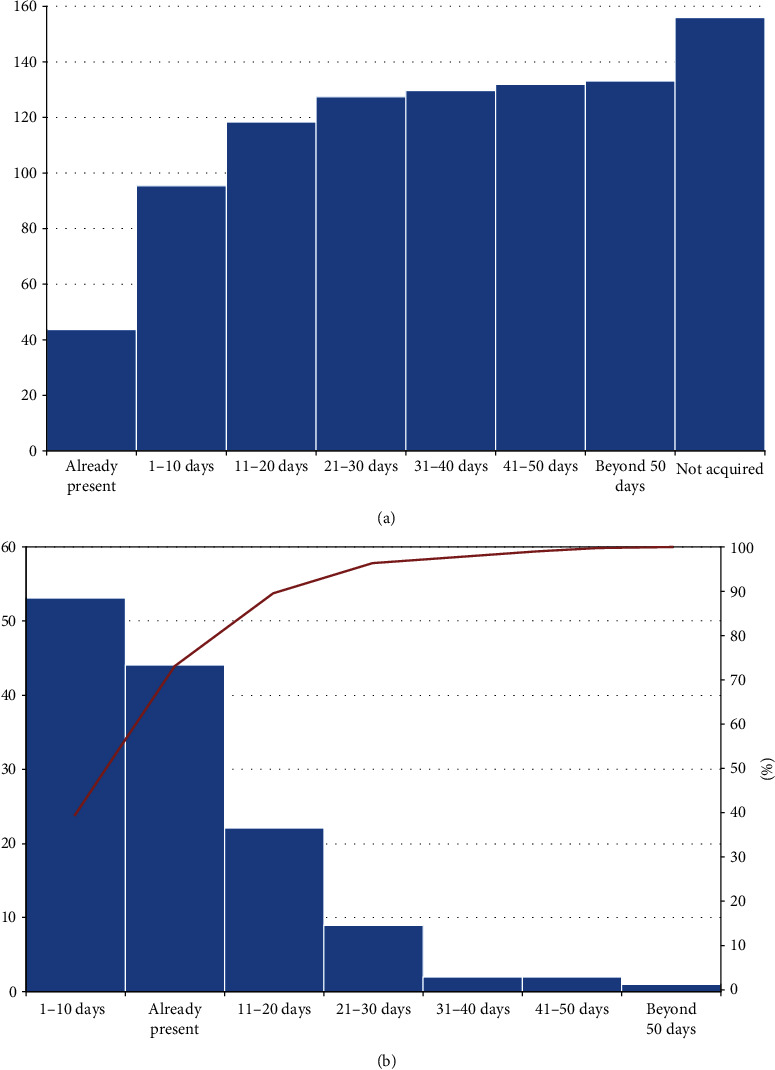
(a) Cumulative frequencies of autonomous standing acquisition times; abscissa, times of milestone acquisition; ordinate, absolute number of patients. (b) Single frequencies of milestone acquisition, ordered according to their frequency; columns, absolute number of patients; line, cumulative percentage.

The recovery timescale of autonomous standing is shown in Figure 2. Nearly 90% of patients could stand alone within 20 days since admission (Figure 1(b)). Multivariable binary logistic regression for standing position at discharge provided a significant model (likelihood ratio: chi square = 50.03, *p* < 0.001). The only variable with a significant relation with the positive outcome (i.e., presence of autonomous standing at discharge) was female sex, however with likely inherent data heterogeneity as shown by large CI (odds ratio, OR, and 95% Confidence Interval (CI): 127.73 [1.02-1590.09], *p* = 0.049).

**Figure 3:**
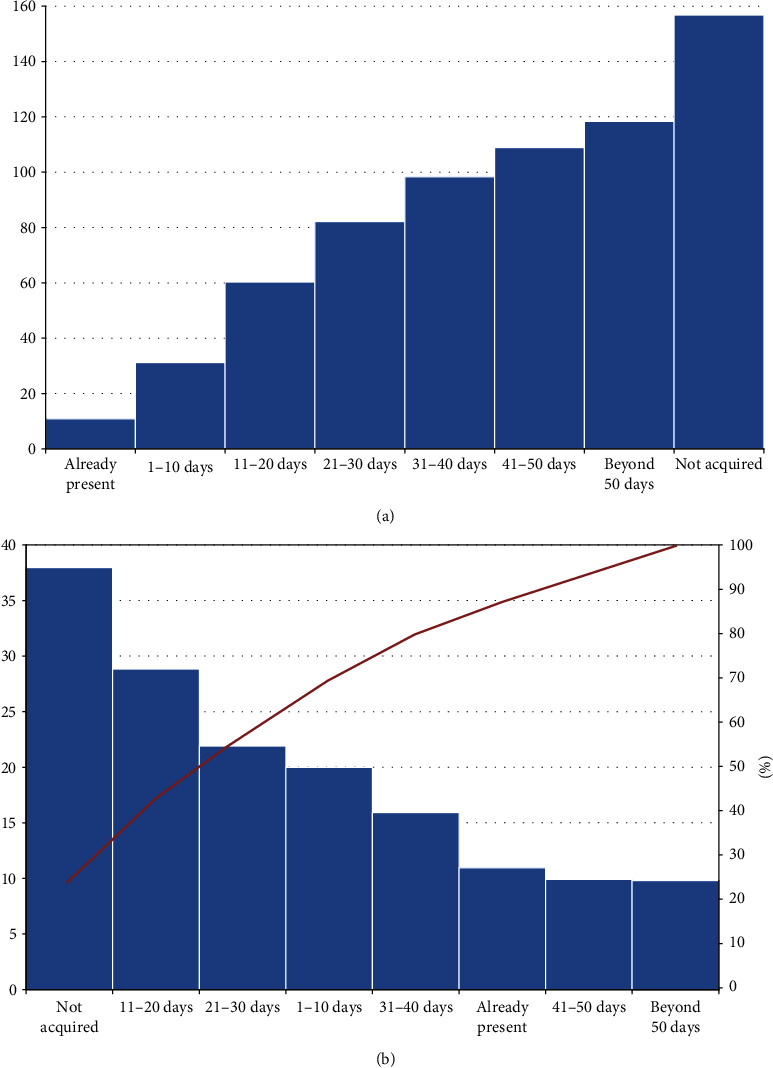
(a) Cumulative frequencies of autonomous walking acquisition times; abscissa, times of milestone acquisition; ordinate, absolute number of patients. (b) Single frequencies of milestone acquisition, ordered according to their frequency; columns, absolute number of patients; line, cumulative percentage.

Figure 3 displays the recovery timescale of autonomous walking. Most of the autonomous walking recovery occurred during the initial 30 days of treatment. Multivariable binary logistic regression for autonomous walking at discharge provided a significant model (likelihood ratio: chi square = 70.51, *p* < 0.001), with two variables significantly related to the outcome: duration of rehabilitative stay (1.05 [1.01-1.09], *p* = 0.012) and Fugl-Meyer score at admission (1.03 [1.01-1.04], *p* = 0.002), although with small odd ratios.

**Figure 4:**
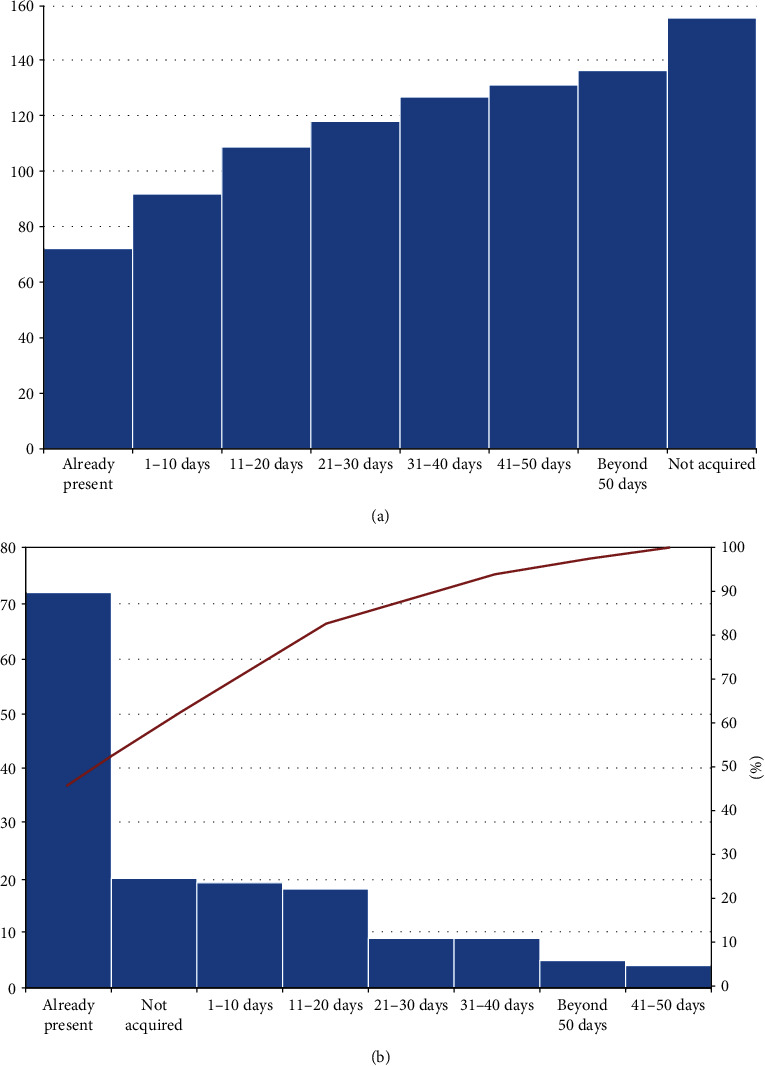
(a) Cumulative frequencies of functional arm acquisition times; abscissa, times of milestone acquisition; ordinate, absolute number of patients. (b) Single frequencies of milestone acquisition, ordered according to their frequency; columns, absolute number of patients; line, cumulative percentage.

The timescale of functional arm recovery (Figure 4) shows that this milestone was less prone to recover (40.9% of patients, Table 2), with the recovery predominantly occurring during the first month of treatment. Multivariable binary logistic regression for the functional arm at discharge also provided a significant model (likelihood ratio: chi square = 57.79, *p* < 0.001), with only one significant association with the outcome, i.e., Fugl-Meyer score at the study entry (1.05 [1.02-1.08], *p* < 0.001).

By stratifying the sampled population according to the quartiles of Fugl-Meyer scores at admission (Table 3), differential patterns of recovery of the main motor milestones were observed, useful to set personalized objective goals and to guide the rehabilitation treatment. Specific, realistic, and attainable outcome goals can be defined for patients classified in different quartiles, and plans can be developed for facilitating proper discharge allocation or for anticipating the need for home adjustment and community support. Patients belonging to the first quartile, for example, who attained autonomous walking in a limited number of cases, can be preferentially addressed by physiotherapic regimes mainly focused on wheelchair mobility. In patients belonging to the higher quartiles, a recovery of the functional arm and autonomous walking can be expected in relatively short times, and tailored measures can be adopted when these milestones do not recover in a timely fashion.

## 4. Discussion

Here, we report on poststroke recovery in a large group of patients affected by a first-ever stroke. All patients showed a variable degree of recovery, with a substantial increase in Fugl-Meyer and FIM scores. Moreover, we traced the time course of recovery of several motor milestones.

The acquisition of three motor milestones, chosen among the others due to their relevance for personal independence in daily life activities, is shown to be significantly associated with female sex (autonomous standing), length of stay and Fugl-Meyer initial score (autonomous walking), and Fugl-Meyer initial score (functional arm), although all these associations explained a relatively small portion of data variability.

### 4.1. Findings from Previous Works and Comparison with the Present Study

The significance of the following comparisons of our findings with those from previous studies is in all cases dampened by (a) the differences in the number of adopted milestones, (b) the lack of detailed and agreed descriptions of the single milestones, (c) the difference in severity of the motor conditions in enrolled patients, which can be only grossly estimated, in part due to the lack of commonly adopted clinical measures, and (d) the difference in many clinical variables affecting the outcome (e.g., time elapsed from stroke to admission and duration of hospital stay). Yet even a tentative comparison may help in understanding the usefulness of the milestone reporting and in correctly selecting the most significant milestones for future studies.

An early work [8] recognized a broadly repetitive pattern of motor and functional recovery following a stroke and reported on time and percentages of acquisition of different milestones in a large group of patients. No correlation analysis was performed, but the paper highlighted the value of collecting the data on recovery milestones for treatment comparisons, to set objective goals and to monitor individualized recovery performances. In that study, four milestones showed a higher rate of unmet acquisition at discharge (maintain sitting balance: 8.2%; stand up to free standing position: 29.3%; independent walking: 46.7%; and place the affected hand to the mouth: 46.2%) compared to the similar ones from our study (Table 2; trunk control: 4.4%, autonomous standing: 27.7%, autonomous walking: 23.9%, and functional recovery of the affected hand: 12.6%). No data are available about the baseline impairment severity in that study.

Another work [9] found that by classifying 95 stroke patients in three subgroups on the basis of their motor, somatic sensory, and visual field deficits, a highly significant difference was obtained in the probability of reaching four milestones (derived by the Barthel Index score: independence in ambulation, ability to walk 150 feet with assistance, independence in self-care function, and reaching a point of assisted care) of poststroke recovery among groups. Patients affected by motor deficits only reached the selected milestones with a higher percentage and more quickly than patients affected by motor and sensory deficits did, and the latter in turn performed better than patients with additional visual field deficits. Lack of raw data and differences in the study setting (the adoption of life table analysis, poorly comparable milestones and clinical categories, and longer observation time) prevent us to compare the reported findings with those from our study.

In another study [10], the recovery time of three milestones (independently reaching a sitting position, independent walk, and stair climbing) was also found to be differentially influenced by four variables (age, perceptual impairments, depression, and comprehension disorders). The only comparable milestone (independent walking) was reached in 46.7% poststroke patients (Table 2: 76.1% in our sample).

Smith and Baer [11] reported on the association between four “mobility milestones” (1-minute sitting balance, 10-second standing balance, a 10-step walk, and a 10-meter walk) and the Oxfordshire Community Stroke Project (OCSP) Classification [21] of cerebral infarction in 238 ischaemic stroke patients. They found the following median recovery times: day of stroke, for the 1-minute sitting balance milestone (recovered in 93% of patients); 3 days, for 10-second standing balance (recovered in 84.7%); 6 days, for 10-step walk (recovered in 80.1%); and 9 days, for 10-meter walk (recovered in 77.7%). Individuals suffering from total anterior circulation infarcts (TACI, according to OCSP classification) achieved the mobility milestones more slowly and had a longer hospital stay, compared to patients affected by partial anterior circulation infarcts (PACI), posterior circulation infarcts (POCI), or lacunar infarcts (LACI). The median values of our comparable milestones were 4 days (trunk control), 9.5 days (autonomous standing), and 22 days (autonomous walking). Overall, the three milestones were reached, at the end of the stay at the hospital, in a comparable percentage of patients (Table 2).

A more recent study [12] collected data on three recovery milestones in a group of 69 patient affected by stroke (both ischaemic or hemorrhagic): the ability to sit without support for longer than 5 minutes, the ability to stand without support in excess of 1 minute, and the ability to walk for 50 meters without assistance. In that cohort of patients, 91% achieved 5-minute sitting balance (median: 7 days), 80% achieved the 1-minute standing balance (median: 8 days), and 59% achieved the 50-meter independent walk (median: 13 days). A combination of the NIHSS score [22] at admission and the presence of posterior cerebral infarct or putaminal hemorrhage predicted achievement of ambulation milestone. Compared to our findings, the three milestones were reached in a slightly lower percentage of patients (Table 2), even though the time of milestone acquisition was similar for trunk control and a little faster for autonomous standing and walking. On the other hand, by comparing the FIM scores at admission in the two samples, our patients showed, on average, a less severe initial functional impairment (Table 1: 64.3 vs. 56.0 FIM score).

### 4.2. Significance of the Study

Acquiring data on the milestones of poststroke recovery has multiple advantages. Firstly, it can provide an outcome set of measures which take into account the temporal dimension of the poststroke pattern of recovery [6, 7]. In addition, it supplies useful and easily recordable information on the degree of patients' functional abilities. As performed in the present study, quantitative data on milestone acquisition can be adopted to detect clinical variables associated with a better recovery [9–12].

Secondly, the knowledge of times of recovery from stroke impairments can guide the management choices, in terms of tailored plans and time of treatment [4, 6]. This is particularly evident whenever it is possible to stratify the patients according to predictive clinical variables from admission, as we suggested by adopting the quartiles of the initial Fugl-Meyer score.

Moreover, percentages and times of milestone acquisition may be used in assessing the effect of alternative treatment regimens, both pharmacological and physical.

Finally, the systematic recording of milestone acquisition can provide a more detailed comprehension of the neurobiology of the poststroke recovery [4, 6, 7]. Poststroke recovery usually exhibits a broadly predictable pattern [4, 8], and a rule of proportional recovery after stroke [7, 23, 24] has been proposed, associated with the initial stroke severity and explaining most of the improvement during poststroke recovery. A subgroup of more severely affected patients, however, does not fit the proportional recovery rule and exhibits much poorer improvements. The temporal pattern of milestone acquisition can help in precociously identifying this subgroup of patients for further in-depth testing and settling specific treatment plans.

Our study shows that the Fugl-Meyer score at admission has a positive association with the acquisition of two functionally relevant milestones, among the selected three. This finding is consistent with the mentioned suggestion that a major predictive variable of poststroke improvement is provided by stroke severity [23, 24]. The finding also highlights the value of the Fugl-Meyer score as a useful clinical measure of neurological impairment.

### 4.3. Limitations and Future Developments

Our study relies on the identification of eight motor milestones during poststroke recovery. It is therefore crucial that milestone description should be extremely detailed, to assure the method reproducibility. Each movement or activity had to be clearly described, easily recognizable, reliably assessed, and scored dichotomously (“absent” or “present”). Unfortunately, no agreement exists in the literature on the milestone selection, nor in the description accuracy of the milestones, which makes it difficult to compare different studies.

The cohort enrolled in the present single-centre study is not representative of all patients affected by stroke, nor the sample size is wide enough to perform correlation measures on all baseline clinical factors. In addition, in some patients, the outcome measures were incompletely collected. These factors, as well as the quite large variability of recovery rates suggested by the relatively small portion of explained data variability and by the relatively small odds ratios, advise that further multicentre studies with a wider case mix are necessary to confirm the present findings and comprehend the possible reasons for heterogeneity in motor recovery.

## 5. Conclusions

This observational study provides quantitative data on reacquisition of motor milestones in a large sample of poststroke patients. It also demonstrates the value of the Fugl-Meyer score in predicting the acquisition of two motor milestones, relevant for daily life activities.

By systematically recording the timescale of motor milestone recovery, along with the Fugl-Meyer score, an improved comprehension of the temporal dimension of the poststroke recovery can be obtained, and a more efficient management of the rehabilitation treatment can be achieved.

## Figures and Tables

**Table 1:** Baseline characteristics of the population study.

	Values
Age (y), mean (SD)	76 (11)
Male sex (%)	53.4
Hypertension (%)	86.9
Carotid atherosclerosis (%)	44.3
Dyslipidemia (%)	43.8
Atrial fibrillation (%)	30.1
Diabetes mellitus (%)	21.6
Chronic ischaemic heart disease (%)	19.9
Depression (%)	17.6
Hyperhomocysteinemia (%)	16.5
Chronic kidney disease (%)	9.7
Barthel Index (%)	57.8 (23.1)
Time from stroke onset to rehabilitation admission (d), mean (SD)	12.0 (14.4)
Type of stroke	
Ischaemic (%)	90.2
Hemorrhagic (%)	9.8
Side of lesion	
Left (%)	51.7
Right (%)	43.1
Bilateral (%)	5.2
Right hemiparesis (%)	52.3
Left hemiparesis (%)	39.2
Aphasia (%)	33.0
Ataxia (%)	24.4
Dysarthria (%)	23.3
Dysphagia (%)	22.2
FIM score at study entry, mean (SD)	64.3 (23.3)
FIM score at discharge, mean (SD)	90.7 (26.4)
Fugl-Meyer score at study entry, mean (SD)	169.8 (41.7)
Fugl-Meyer score at discharge, mean (SD)	191.8 (41.7)
Length of stay (d)	52.6 (18.2)

**Table 2:** Number (and %) of patients who reached the recovery milestones during the observation period and mean days (and SD) elapsed after admission for recovery of each milestone (data available for 159/176 patients).

Milestone	Already present at admission	Not acquired at discharge	Acquired during treatment	Mean time of recovery
Muscle tone recovery	137 (86.2)	5 (3.1)	17 (10.7)	6.8 (5.6)
Trunk control	125 (78.6)	7 (4.4)	27 (17)	6.9 (10.7)
Assisted standing	111 (69.8)	8 (5)	40 (25.5)	8.0 (10.7)
Autonomous standing	44 (27.7)	23 (14.5)	92 (57.9)	12.4 (10.8)
Assisted walking	73 (45.9)	15 (9.4)	71 (44.7)	10.9 (11.6)
Autonomous walking	11 (6.9)	38 (23.9)	110 (69.2)	25.9 (16.3)
Active motility of the affected arm	128 (80.5)	12 (7.5)	19 (11.9)	13.5 (16.3)
Functional recovery of the affected arm	74 (46.5)	20 (12.6)	65 (40.9)	21.8 (16.1)

**Table 3:** Percentage of retained and recovered milestones as a function of Fugl-Meyer scores at admission.

Fugl-Meyer score at admission (quartiles)	AS		AW		FA	
	adm	dis	adm	dis	adm	dis
0-145 (I)	2.5	62.5	2.5	42.5	27.5	60.0
146-184 (II)	18.0	89.7	5.1	74.4	35.9	89.7
185-206 (III)	38.9	97.2	5.6	94.4	47.2	100
207-255 (IV)	51.3	94.4	13.9	94.4	77.8	100

AS: autonomous standing; AW: autonomous walking; FA: functional arm; adm: admission; dis: discharge.

## Data Availability

The clinical data that support the findings of this study are available from the corresponding author upon reasonable request.
